# A comprehensive analysis of the prognostic characteristics of microRNAs in breast cancer

**DOI:** 10.3389/fgene.2024.1293824

**Published:** 2024-03-20

**Authors:** Lingying Wang, Gui Wang, Jiahong Song, Di Yao, Yong Wang, Tianyou Chen

**Affiliations:** ^1^ Department of Thoracic Surgery, Wuhan Third Hospital, Tongren Hospital of Wuhan University, Wuhan, China; ^2^ Department of General Surgery, The Second Affiliated Hospital of Anhui Medical University, Hefei, China

**Keywords:** breast cancer, overall survival, disease specific survival, microRNA-551b, cancer progression

## Abstract

Both overall survival (OS) and disease-specific survival (DSS) are significant when determining a patient’s prognosis for breast cancer (BC). The effect of DSS-related microRNAs on BC susrvival, however, is not well understood. Here, we spotted differentially expressed miRNAs (DEMs) in the TCGA database of BC DSS, identified eight DSS-related miRNAs, and constructed a risk model. AUC values at 1, 3, and 5 years were 0.852, 0.861, and 0.868, respectively, indicating a risk model’s excellent prognostic prediction ability. Then, we validated miRNA roles in BC OS and finally defined miR-551b as an independently prognostic miRNA in BC. According to function analysis, miR-551b is strongly linked with the emergence and spread of cancer, including protein ubiquitination, intracellular protein transport, metabolic pathways, and cancer pathways. Moreover, we confirmed the low expression of miR-551b in BC tissue and cells. After miR-551b inhibition or overexpression, cell function was either dramatically increased or diminished, respectively, indicating that miR-551b could regulate BC proliferation, invasion, and migration. In conclusion, we thoroughly clarified BC-related miRNAs on DSS and OS and verified miR-551b as a crucial regulator in the development and prognosis of cancer. These results can offer fresh ideas for BC therapy.

## 1 Introduction

Breast cancer (BC) is known as the most common malignancy among women, with the second-highest mortality rate in the world (Siegel et al., 2021). Although the 5-year survival rate of BC is very high, the survival period will be greatly shortened if the patient has metastasis or recurrence (Wang et al., 2019). Overall survival (OS) is relatively convenient for assessing BC survival and is the most common index for prognostic assessment. However, it cannot exclude the influence of non-tumor related deaths (Gourgou-Bourgade et al., 2015). The disease specific survival (DSS) was defined as patients who died from a specific disease in a period (Montazeri et al., 2016). Targeted response to clinical benefit, DSS enhancement can well reflect the clinical benefit of specific diseases, and the death caused by specific diseases is reduced or increased (Liu et al., 2018). Although the OS shows significance in the prognosis assessment of cancer patients, DSS is also indispensable, which enables our comprehensive monitoring of cancer development and deterioration to timely intervention and treatment. However, the role of DSS in BC has been less studied.

Non-coding RNA gene sequences make up over 98% of the human genome, whereas coding RNA gene sequences make up only 2%, indicating that protein transcription is tightly regulated (Esteller, 2011; Iorio and Croce, 2012; Morris and Mattick, 2014). MicroRNAs (miRNAs) were defined as highly conserved non-coding RNAs about 20–24 nucleotides in length (Lee and Dutta, 2009; Hombach and Kretz, 2016). Even though miRNAs cannot code for proteins, miRNAs are deeply involved in biological processes via their downstream gene. The mechanism of miRNA function is to form RNA induced Silencing complex (RISC) and result in the degradation of target gene, namely, Post-transcriptional Gene Silencing (PTGS) (Tiwari et al., 2021). As a result, miRNAs can regulate all aspects of tumor formation, cancer progression, and distant metastasis, especially in breast cancer (He et al., 2020). Many reporters have confirmed miRNA roles in BC prognosis, cancer proliferation, invasion, angiogenesis, and metastasis (Bertoli et al., 2015; Xu et al., 2020). Among them, some miRNAs function in BC cell adhesion, including has-miR-33a, has-miR-198, has-miR-155, has-miR-21, and has-miR-10b, *etc.*,; Some miRNAs involved in tumor microenvironment (TME), for example, has-miR-593, has-miR-494, has-miR-320, has-miR-193b, has-miR-181a, and has-miR-29b, *etc.*,; Some miRNAs promote breast cancer stemness, including has-miR-183 has-let-7b, has-miR-140, has-miR-221, and has-miR-30 (Fridrichova and Zmetakova, 2019; Flores-Huerta et al., 2021). MiRNAs that serve as therapeutic targets for breast cancer, such as has-miR-339-5p, has-miR-187, and has-miR-30c. These studies indicate that miRNAs have powerful efforts in regulating BC biological processes, so it is of great importance to explore the role of miRNAs in BC prognosis and progression in a comprehensive analysis.

In this study, through the breast cancer TCGA database, we comprehensively analyzed the expression and prognostic characteristics of DSS-related miRNAs in BC. By constructing a prognostic model, we explored its prognostic predictive ability in BC survival. The OS and DSS related miRNAs and their prognostic relationships were further identified, and miR-551b was identified as a prognosis miRNA of endpoint events. We further validated miR-551b expression in BC tissues and cells and revealed its effect on cancer proliferation, and invasive migration.

## 2 Materials and methods

### 2.1 Data selection and processing

The cohort of the TCGA Breast Cancer (TCGA_BRCA) dataset, which contains a total of 1,202 samples, including 1098 BC samples and 104 normal samples, was downloaded from the official UCSC website (Goldman et al., 2020). Next, we annotated, normalized, and standardized the data and calculated differentially expressed miRNAs (DEMs) (Love et al., 2014). DEMs that met the screening criteria were defined as log2 fold change (log2 FC) greater than 0.5, with a *p*-value of less than 0.05, and chosen for further validation. The overall survival (OS) and disease specific survival (DSS) data were obtained from “survival data” (Weinstein et al., 2013). By organizing the data, we deleted incomplete data and cases with survival times of less than 15 days and obtained a total of 889 patients’ survival outcomes. These cases will be used for subsequent analysis.

### 2.2 Prognostic modeling

We first calculated the hazard ratios (HR) of each DEM in the TCGA DSS group (training group), and defined a *p*-value less than 0.05 as the screening condition. The miRNAs in the training group that met these screening criteria were identified as prognostic miRNAs, and their expression and prognosis were validated in the TCGA_BRCA dataset. Additionally, we incorporated variables from the multivariate Cox analysis and constructed a prognostic risk model. Only miRNA with *p*-value less than 0.05 were included in multivariate Cox analysis.

The formula of risk score can be calculated in X1α1 + X2α2 + X3α3 +…+ Xnαn. Then, we categorized patients into high-risk groups and low-risk groups based on median scores (Park, 2018). Also, we calculated the AUC values of the 1-, 3-, and 5-year ROC curves to evaluate the risk model’s capacity to predict outcomes, and the Kaplan-Meier analysis was used to compare the differences in overall survival. Then, we constructed a nomogram to intuitively display the weight of each DEM in the risk model. The value between the model-predicted survival and the actual one was assessed by the corrected curves, and the survival value of the miRNAs was verified in the KM-plotter database. Due to the lack of additional DSS databases, we split the TCGA_BRCA database evenly and randomly into two databases and used one of them as the validation dataset. The prognosis of DSS-related DEMs was assessed by validation dataset through Cox analysis, survival analysis and ROC curves. Similarly, we validated the prognostic effect of DEMs in overall survival (OS) and defined as TCGA_OS group. The prognosis of OS-related DEMs was confirmed by univariate Cox analysis, Lasso regression, and multivariate Cox analysis separately. Finally, we confirmed the prognostic effect of DEMs in all three databases including the TCGA_DSS group, TCGA_OS group, and DSS validation group, and selected DEMs with differences for subsequent analysis.

### 2.3 Functional validation of miR-551b

We found the downstream genes of has-mir-551b (miR-551b) through TargetScan (Agarwal et al., 2015) and miRNet (Chang et al., 2020) database, respectively, and took the genes common to both databases as their target genes. Then we explored the functions of target genes in the Davaid database (Huang da et al., 2009) and KOBAS database (Bu et al., 2021), including the gene ontology (GO) function and KEGG (Kyoto Encyclopedia of Genes and Genomes) pathway (Kanehisa and Goto, 2000), where the GO function including GO_BP (Biological Process), GO_CC (Cellular Component), GO_MF (Molecular Function) (Maag, 2018). The functions and potential pathways of miR-551b enrichment were mapped by “ggplot2” package (Gustavsson et al., 2022).

### 2.4 Tissue acquisition and cell culture

Twelve pairs of breast cancer and its paracancerous fresh tissues were collected from Wuhan Third Hospital in 2021 and preserved in liquid nitrogen. Postoperative pathology confirmed the diagnosis of breast cancer, and none of them had been treated. This study was admitted by the Ethics Committee of the Third Hospital of Wuhan City (WQ20210274). We bought the normal breast epithelial cell line (MCF-10A) from Procell Biotechnology, while the BC cell lines SKBR3, T47D, MCF-7, MDA-MB-231, and MDA-MB-468 were purchased from the cell bank of the Chinese Academy of Sciences. Cells were cultivated in a cell incubator at 37°C with 5% CO2 using the following media: MCF7, MDA-MB-231, and MDA-MB-468 were cultured in DMEM; SKBR3 was cultured in McCoy’s 5A medium; T47D was cultured in RPMI-1640 medium; and MCF-10A was cultured in DMEM/F12 medium, all media were added with 10% fetal bovine serum.

### 2.5 Real-time quantitative polymerase chain reaction experiments

Using the TRIzol reagent, total RNA was extracted from tissues and cells separately. Nano 50 was used to measure the concentration and purity of the total RNA. The cDNA was synthesized in a two-step process using the Reverse Transcriptase Kit (Novozymes, MR101-01) according to the instructions. Afterwards, a cDNA was produced under the following reaction conditions: reaction at 42°C for 2 min, followed by cooling to room temperature. The ABI 7500 was used to carry out the PCR quantitative amplification reaction under the following conditions: 25°C for 5 min, 50°C for 15 min, and 85°C for 5 min. The following ingredients were used in the amplification system: 2 μL cDNA, 0.4 μL mQ primer, 2 μL specific primer, 10 μL SYBR, and finally, ddH2O to make 20 μL total. Amplification conditions were performed according to the instruction manual (Novozymes, MQ101-01). We set the U6 as the endogenous reference, used the fusion curve to determine the primer specificity, and calculated the relative expression according to the 2–ΔΔ CT method. The following sequences were synthesized by Sangon biotech to create the primers: U6: 5' -CGC AAG GAT GAC ACG CAA AT-3' (Forward), 5′-CGG CAA TTG CAC TGG ATA CG-3' (Reverse). Has-miR-551b-5p (miR-551) has the sequences 5′- CGG AAA TCA AGC GTG GGT-3' (forward) and 5′- AGT GCA GGG TCC GAG GTA TT-3' (reverse). RT Primer: 5′- GTC GTA TCC AGT GCA GGG TCC GAG GTA TTC GCA CTG GAT ACG GTC TC-3'.

### 2.6 Cell transfection and function verification experiments

The miR-551b mimics (agomiR-551b) and inhibitor (antagomiR-551b) were designed and synthesized by Suzhou Gemma Biologicals and transfected as instructions. In 6-well plates, prepared cells were counted and planted. Separate mixtures of the diluted Lipo 3,000 and miR-551b were applied to 6-well plates at cells in 70% confluence. After 20 min reaction at room temperature, cells were continued culture and chosen for subsequent experiments.

For cell proliferation assay, transfection cells in satisfactory development were digested and injected into 96-well plates for the CCK-8 studies. 10 μL of CCK-8 reagent was added to each well at 0, 24, 48, and 72 h after the cells had been in for 2 h. Subsequently, in preparation for further study, we estimated the absorbance of cells at 450 nm.

The matrix gel was first applied to the Transwell chamber and left there to solidify. In the upper chamber, we added a medium devoid of 10% fetal bovine serum (FBS), while in the lower chamber, we added a medium with 10% FBS. Then, cells were counted, put into the upper chamber, and incubated for a total of 24 h. After being removed from the top chamber, the cells were stained using hematoxylin-eosin, fixed using 4% paraformaldehyde, and numbered beneath microscope.

The wound healing experiment went through the following steps. A 6-well plate was inoculated with cells that had been developed throughout the growth phase. After the cell confluence reached 70%, we then scraped the cells with a 200 L sterile tip. Microscopically, cell scratches changed in width at 0 and 24 h. The cell migration rate was estimated for further investigation.

### 2.7 Statistical analysis

The data was examined and processed using R and GraphPad Prism 8.0. For comparing two groups, we utilized a t-test, and for analyzing multiple groups, we used a one-way ANOVA. Kaplan-Meier was used to compute the cumulative survival rate, and the log-rank t-test was employed for statistical analysis. Every experiment was carried out thrice, and results were deemed as statistically significant at P 0.05.

## 3 Results

### 3.1 Identification of DSS-related miRNAs in breast cancer

**FIGURE 1:**
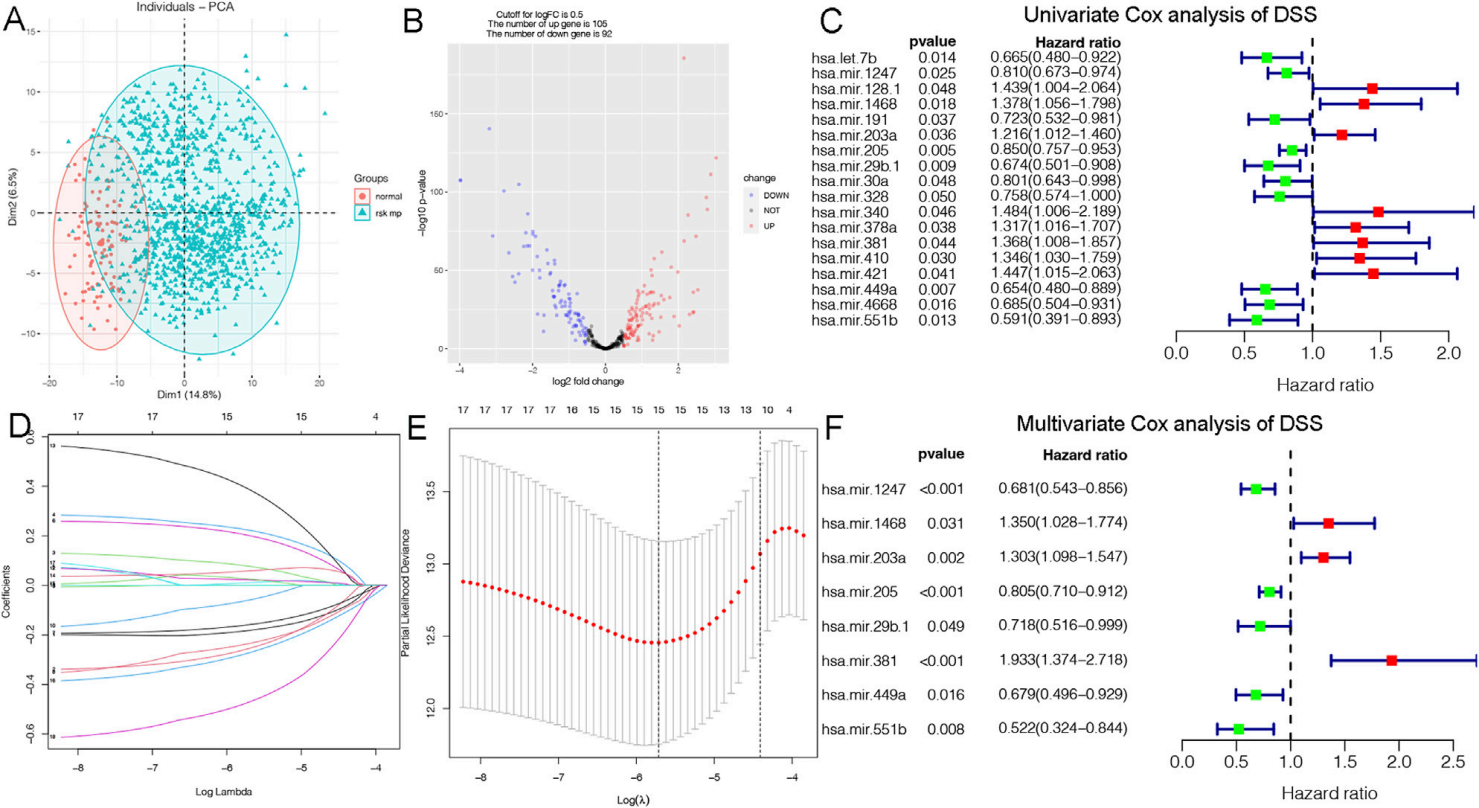
Identification of DSS-related DEMs of breast cancer (BC). **(A)** PCA demonstrating the distribution of tumor patients and normal patients in the TCGA_BRCA database; **(B)** Volcano plot showing the differentially expressed miRNAs (DEMs) of disease specific survival (DSS); **(C)** Univariate Cox analysis validating the prognostic effect of DSS-related DEMs in BC; **(D, E)** Lasso regression to optimize the univariate results and include the best variables into the multivariate analysis; **(F)** Multivariate Cox analysis confirming qualified DEMs for risk model, in which hazard ratio (HR) greater than 1 is a risk factor, and HR less than 1 is protective, with *p*-value < 0.05 as the screening threshold.

**FIGURE 2:**
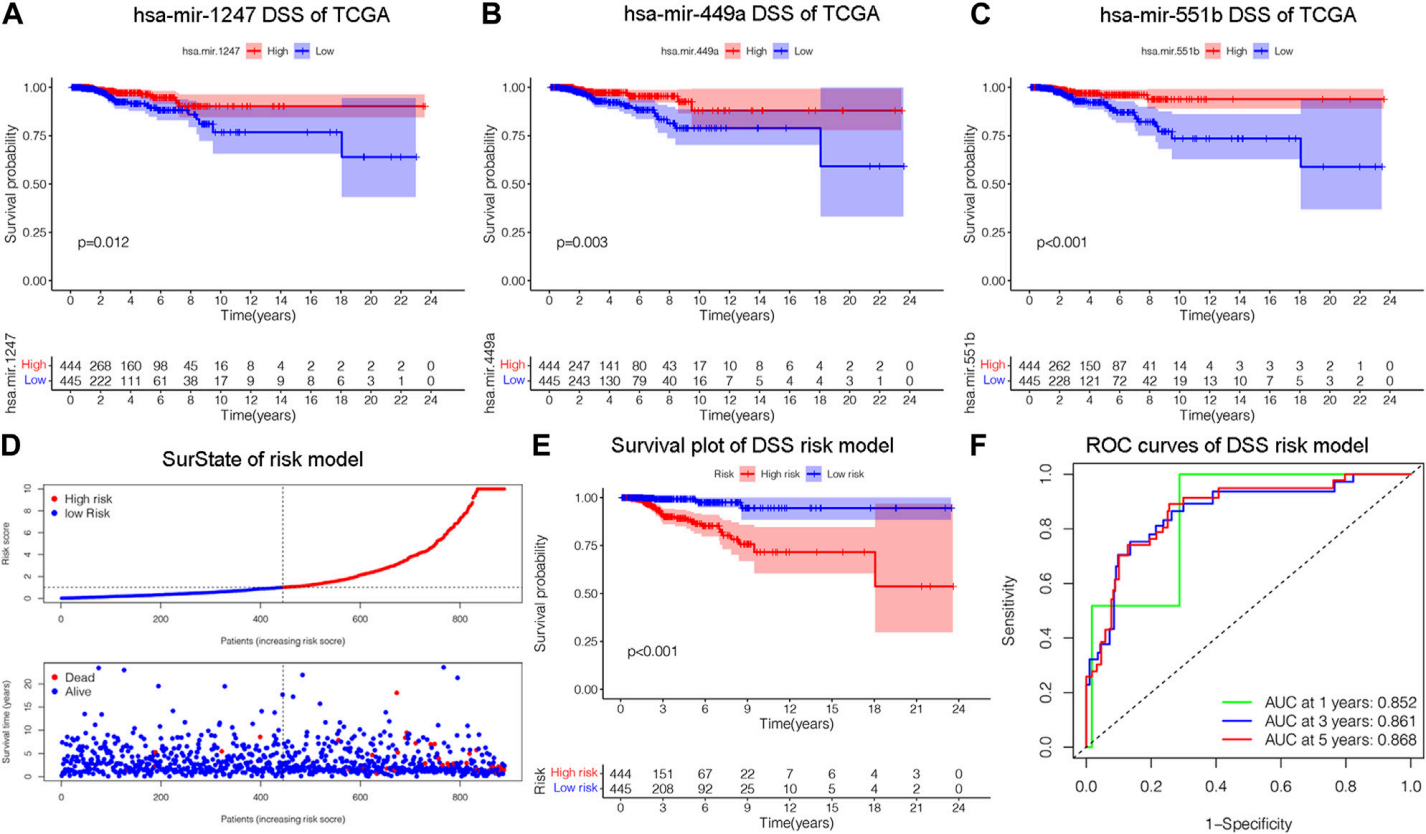
Construction of a risk model related to disease specific survival (DSS). **(A–C)** Validating the prognostic effect of DSS-related DEMs, including hsa-mir-1247, hsa-mir-449a, hsa-mir-551b, in the TCGA database, respectively. **(D)** The risk model classified BC patients into high and low-risk groups according to DSS score. **(E)** KM survival analysis assessed the difference in survival between high- and low-risk patients. **(F)** ROC curves validated the predictive ability of the risk model at 1, 3, and 5 years, respectively.

Disease specific survival (DSS) well reflects the clinical benefit of specific diseases. But Less is known about the role of DSS in BC miRNAs. In this research, we identified miRNAs related to DSS and OS. Through TCGA_DSS, TCGA_OS, AND DSS validation groups, we confirmed the protective factor of miR-551b in BC prognosis. This study included a total of 1,202 samples, including 104 normal samples and 1,098 breast cancer samples. We categorized data as differentially expressed miRNAs (DEMs) if log2FC was larger than 1 and *p*-value was less than 0.05. PCA plot revealed a distinct distribution between patients with tumors and healthy people (Figure 1A). The TCGA_BRCA dataset had 197 DEMs in total, 105 of which were upregulated and 92 of which were downregulated, and a volcano plot of the DEMs was demonstrated (Figure 1B). Furthermore, we obtained a total of 889 patients with complete follow-up data, and chose for subsequent analysis. Our results identified 18 meaningful DSS DEMs in univariate Cox analysis (Figure 1C). To increase the reliability of multivariate results, we further analyzed their survival by Lasso analysis. As displayed in Figures 1D, E, the Lasso analysis recognized 15 DEMs that qualified for multivariate models. Next, the multivariate Cox regression indicated a total of eight prognosis miRNAs, of which, has-mir-1247 had an HR and 95% CI of 0.681 (0.543–0.856) with a *p*-value of 0.001 in the multivariate Cox analysis. The hsa-mir-1468 had an HR and 95% CI of 1.350 (1.028–1.774) and a *p*-value of 0.031. The has-mir-203a had a *p*-value of 0.002 and an HR and 95% CI of 1.303 (1.098–1.547). The hsa-mir-205 had an HR and 95% CI of 0.805 (0.710–0.912) and a *p*-value of 0.001. With a *p*-value of 0.049, the has-mir-29b.1 had an HR and 95% CI of 0.718 (0.516–0.999). The has-mir-381 had an HR and 95% CI of 1.933 (1.374–2.718), with a *p*-value of 0.001. The has-mir-449a had a *p*-value of 0.016 and an HR and 95% CI of 0.679 (0.496–0.929). The HR and 95% CI of hsa-mir-551b was 0.522 (0.324–0.844) with a *p*-value of 0.008 (Figure 1F). Additionally, we validated the expression and prognosis of 8 DEMs in TCGA_DSS group with *p*-value screening criteria. The findings revealed that high expression of hsa-mir-381 and hsa-mir-410 had a lower survival time in breast cancer, while hsa-mir-1247, hsa-mir-449a, hsa-mir-551b, hsa-let-7b, hsa-mir-205 expression levels were associated with a longer survival time (Figure 2). Finally, we validated 7 DEMs related to DSS outcome (Figures 2A–C; Supplementary Figures S1A–D), and chose for further analysis.

### 3.2 Constructing a prognostic model on DSS

We constructed a prognostic model and evaluated its effect on the DSS by ROC analysis, survival analysis and calibration curve. Initially, we evaluated the prognosis of each DEM in BC DSS via univariate Cox analysis. Eighteen DSS-related DEMs were identified with *p*-value less than 0.05. To assess the qualified DEMs in multivariate analysis, we removed DEMs that may be highly intercorrelated via Lasso Cox analysis to avoid overfitting, which could confound the prediction results. As displayed in Figures 1D, E, there are 15 DEMs qualified in multivariate analysis. In multivariate Cox analysis, the risk score of each patient was evaluated. And we assigned each patient a prognostic risk score and divided them into high-risk and low-risk groups. Eight DEMs were related to DSS of breast cancer both in univariate and multivariate Cox analysis. Then, we developed a risk model with formula as follows: (−0.38*hsa-mir-1247) + (0.30*hsa-mir-1468) + (0.26*hsa-mir-203a) + (−0.21*hsa-mir-205) + (−0.33*hsa-mir-29b.1) + (0.66*hsa-mir-381) + (−0.39*hsa-mir-449a) + (−0.65*hsa-mir-551b). The risk model was able to distinguish between patients with high-risk and low-risk conditions, according to the model distribution of death and risk (Figure 2D). With a *p*-value of 0.01, the risk model significantly differences in survival results between the high-risk group and low-risk group, indicating an excellent DSS predictive capacity (Figure 2E). The ROC curve results revealed that the model’s predictive power at 1, 3, and 5 years was 0.852, 0.861, and 0.868, respectively (Figure 2F). Additionally, a nomogram risk model was constructed based on the expression of prognostic DEMs, which could predict BC prognosis at 1, 3, 5 years (Supplementary Figure S1E). The calibration curves showed that the model’s capacity to predict for one, three, and 5 years was compatible with the actual OS prognosis taking place (Supplementary Figures S1F–H). These findings imply that our risk model can accurately determine the DSS prognosis for BC.

### 3.3 Validation the overall survival of prognostic model

**FIGURE 3:**
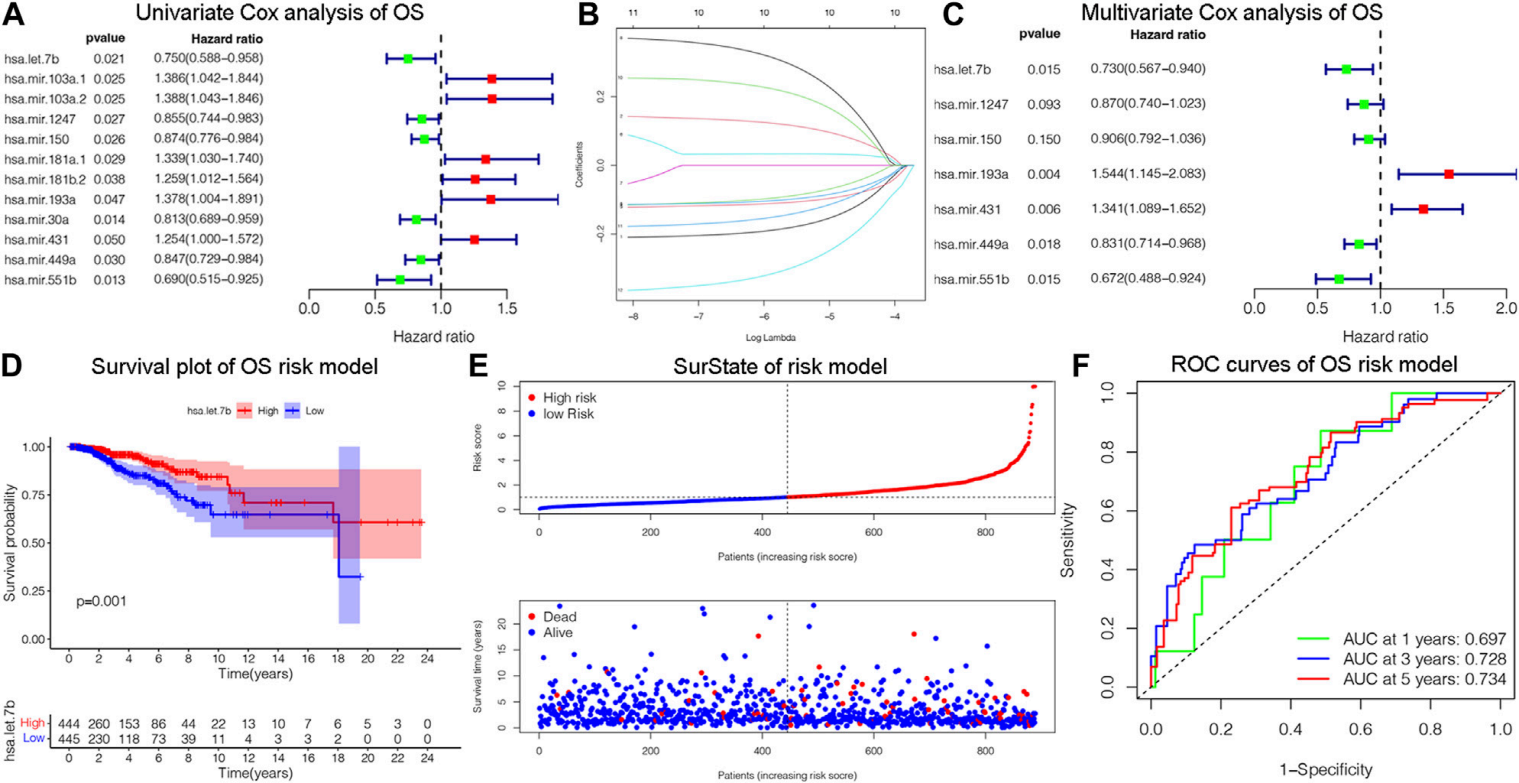
Identification of OS-related DEMs of breast cancer (BC). **(A)** Univariate Cox analysis validating the prognostic effect of OS-related DEMs in BC; **(B)** Lasso regression optimizing the univariate results and including the best variables into the multivariate analysis; **(C)** Multivariate Cox analysis confirming qualified DEMs for risk model. **(D)** KM survival analysis assessed the difference in survival between high- and low-risk patients. **(E)**The risk model classified BC patients into high and low-risk groups according to OS score. **(F)** ROC curves validated the predictive ability of the risk model at 1, 3, and 5 years, respectively.

**FIGURE 4:**
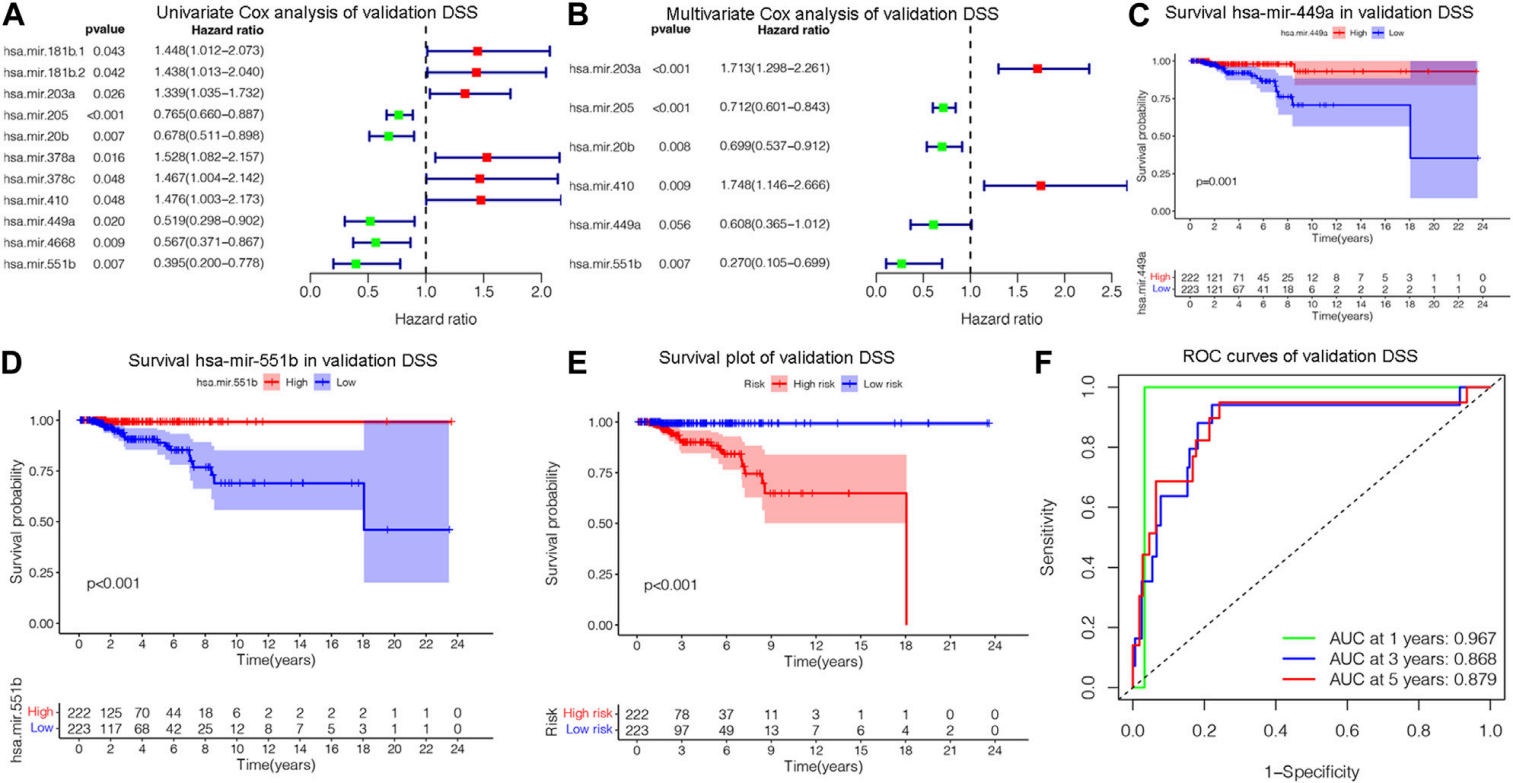
Assessing DSS-related DEMs in the validation group. **(A)** Univariate Cox analysis validating the prognostic effect of DSS-related DEMs in the validation group; **(B)** Multivariate Cox analysis confirming qualified DEMs for risk model. **(C, D)**Validating the prognostic effect of hsa-mir-1247, hsa-mir-449a, and hsa-mir-551b in the validation group. **(E)** KM survival analysis assessed the difference in survival between high- and low-risk patients. **(F)** ROC curves validated the predictive ability of the risk model at 1, 3, and 5 years, respectively.

The OS is an important prognostic outcome of BC. We assessed the OS-related miRNAs using univariate Cox regression, Lasso regression, and multivariate Cox regression, respectively. As shown in Figures 3A–B, there are 12 OS-related DEMs in univariate analysis, and 10 DEMs qualified in multivariate analysis. 5 DEMs were significant in multivariate analysis and enrolled in risk model (Figure 3C). The has-let-7b had an HR and 95% CI of 0.730 (0.567–0.940) with a *p*-value of 0.015 in the multivariate Cox analysis. The hsa-mir-193a had an HR and 95% CI of 1.544 (1.145–2.083) and a *p*-value of 0.004. The has-mir-431 had a *p*-value of 0.006 and an HR and 95% CI of 1.341 (1.089–1.652). The has-mir-449a had a *p*-value of 0.018 and an HR and 95% CI of 0.831 (0.714–0.968). The HR and 95% CI of hsa-mir-551b was 0.672 (0.488–0.924) with a *p*-value of 0.015. Subsequently, using ROC analysis, survival analysis, and calibration curve, we created a risk model and assessed its impact on OS (Figures 3D–F). With a *p*-value of 0.01, the risk model dramatically different survival outcomes from the high-risk group and low-risk group (Figure 3D). The prognostic model was able to distinguish between individuals who had high- and low-risk conditions, based on the model distribution of death and risk (Figure 3E). ROC curves demonstrated that the OS model’s accuracy of prediction at 1, 3, and 5 years were 0.697, 0.728, and 0.734 separately (Figure 3F). Additionally, we verified the OS of 18 DSS-related miRNAs. As depicted in Supplementary Figure S3, 5 DEMs were identified in univariate analysis and 3 qualified DEMs in multivariate analysis. And the risk model was excellent in survival identification and ROC diagnosis with 1, 3, and 5 years of 0.654, 0.703, and 0.687. Due to the lack of additional DSS databases, we split the TCGA_BRCA database evenly and randomly into two databases and used one of them as the validation group. There are a total of 445 patients enrolled in the validation group. We verified DSS-related DEMs in the validation group by Cox regression, Lasso regression, and risk model construction. As displayed in Figures 4A, B, there are 11 DEMs in univariate analysis, and 5 DEMs qualified in multivariate analysis, including has-mir-203a, hsa-mir-205, hsa-mir-20b, hsa-mir-410, hsa-mir-551b without hsa-mir-449a. We first validated their roles in prognosis (Figures 4C, D) and then constructed a risk model, and verified the roles of survival and their diagnosis in DSS validation group (Figures 4E–F). Inspiringly, the risk model is not only significant in survival distinction, but also superior in ROC diagnosis with 1, 3, and 5 years of 0.967, 0.868, and 0.879. Those results indicated that our model was proficient in DSS and OS prognosis. As mentioned above, hsa-mir-449a and hsa-mir-551b were excellent in DSS and OS recognition. Only hsa-mir-551b shown significance with *p*-value less than 0.05 among three groups including TCGA_DSS, TCGA_OS, and DSS_validation. Except for our databases, we proved their survival in KM Plotter database, which have four miRNA databases including METABRIC, TCGA, GSE40267, and GSE19783 datasets. So we validated the survival roles of hsa-mir-449a and hsa-mir-551b. As shown in Supplementary Figure S4, both hsa-mir-449a and hsa-mir-551b have survival significance in METABRIC and TCGA datasets, which fit with our previous results. Altogether, Only hsa-mir-551b showed significance among three groups. So we confirmed hsa-mir-551b both related to DSS and OS outcome and chosen for further analysis.

### 3.4 Functional validation results

**FIGURE 5:**
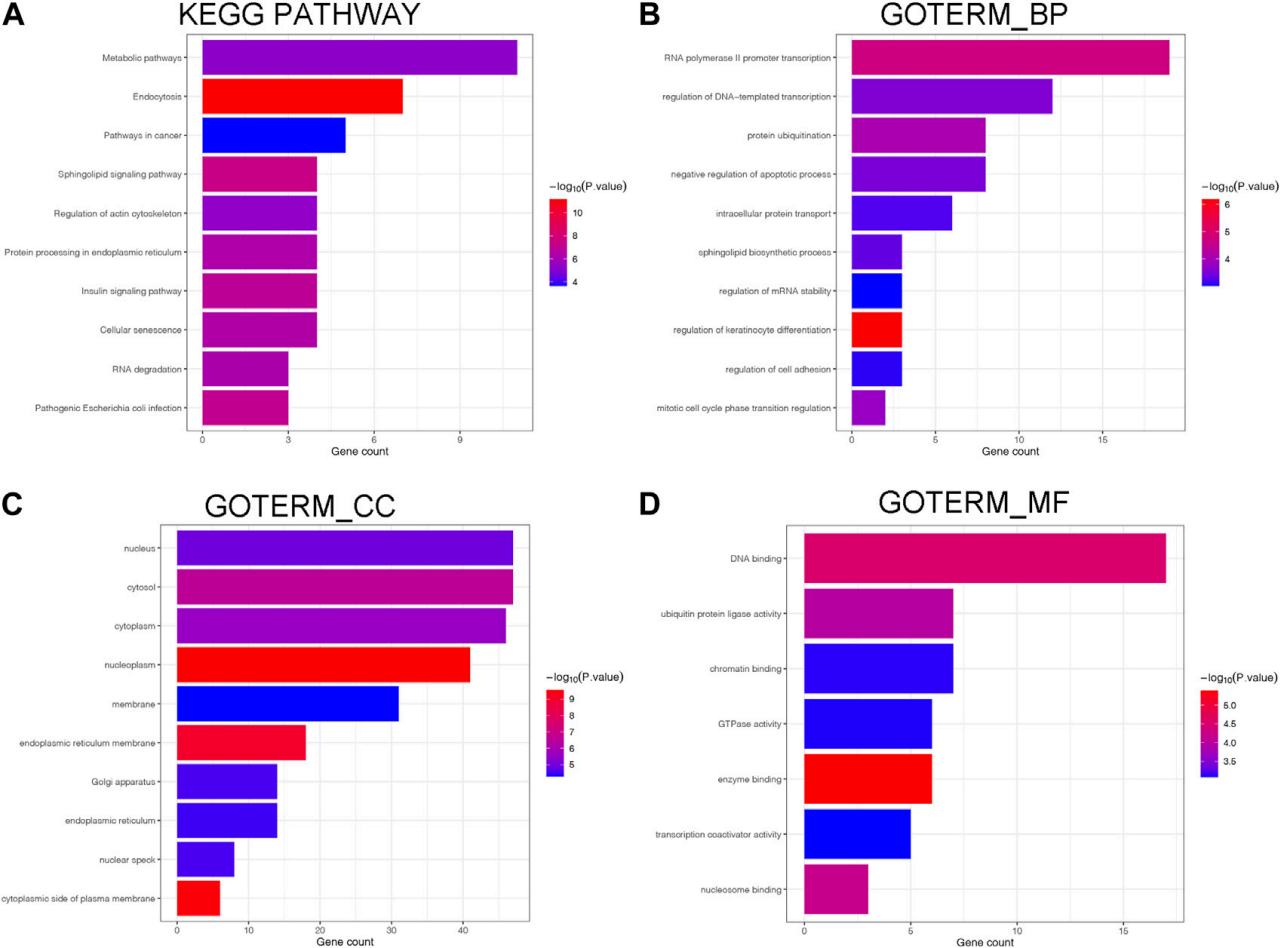
Biological processes and enrichment pathways involved in miR-551b. **(A)** The bar plot of top10 KEGG pathway in miR-551b enrichment; **(B–D)** The bar plot of top10 GO terms in miR-551b enrichment, including GO_BP (Biological Process), GO_CC (Cellular Component), GO_MF (Molecular Function). The number of gene counts represents correlation with the pathway, and log-transformed *p*-values mean significance.

As depicted in Figure 5, we annotated the functional role of miR-551b. Targetscan and TarBase databases revealed that miR-551b has a total of 124 target genes. Then, we explored the functions of target genes in the Davaid database and KOBAS database with a cutoff criterion of *p* < 0.05. Next, top ten most enriched GO terms and KEGG pathways were displayed. We provided all the functional results in Supplementary Table S3, and displayed top ten most enriched GO terms and KEGG pathways in Figure 5. As the DAVAID analysis demonstrated, miR-551b was engaged in several biological processes in malignancies, for example, protein ubiquitination, intracellular protein transport, regulation of mRNA stability, endoplasmic reticulum membrane, enzyme binding, and chromatin binding. It is also linked to a variety of pathways that are associated with tumors, including RNA degradation, insulin signaling, metabolic pathways, cancer pathways, sphingolipid signaling, and pathways for insulin and cancer. These findings imply a link between the miR-551b and the emergence of cancer.

### 3.5 miR-551b regulating BC progression

**FIGURE 6:**
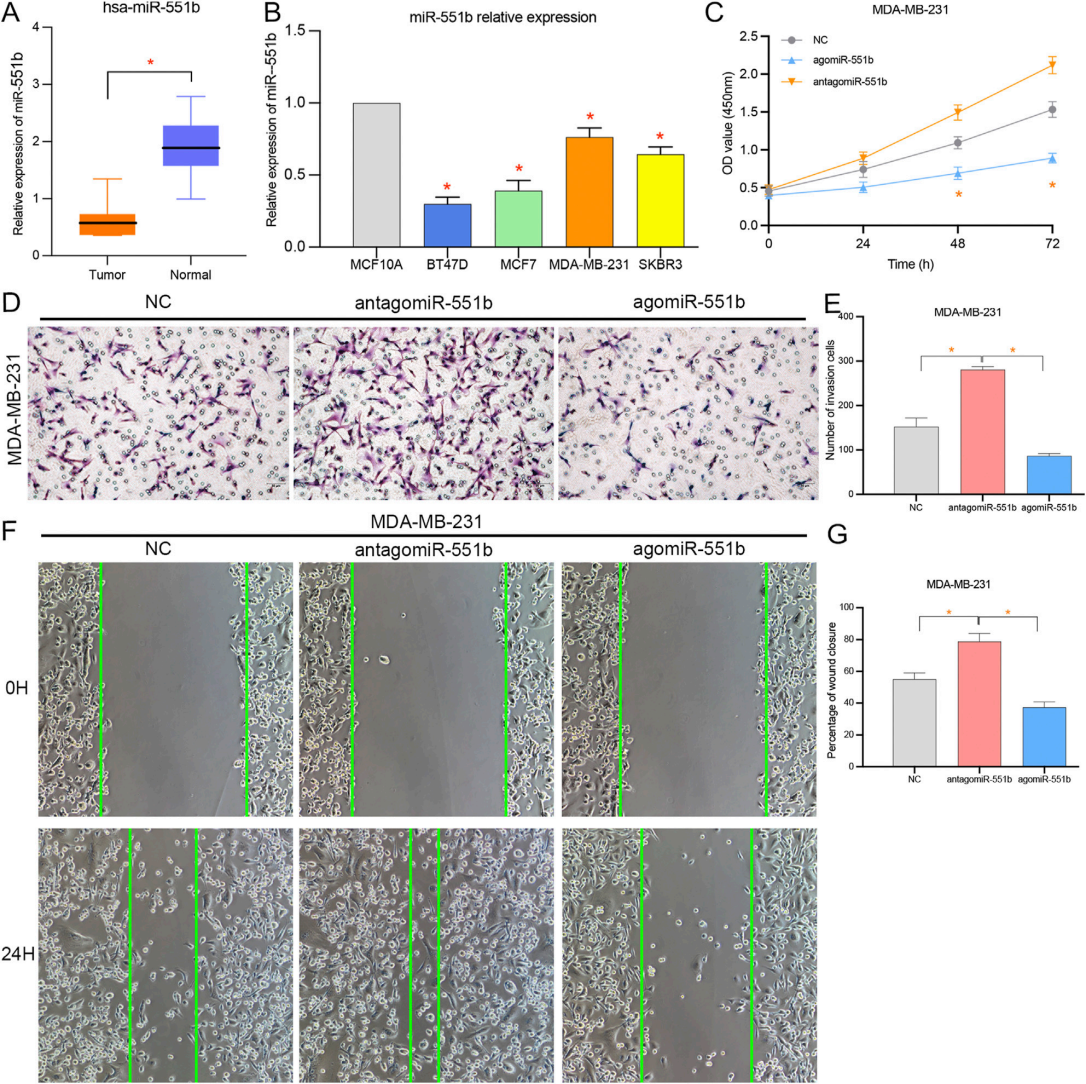
Effects of miR-551b on the proliferation, invasion, and migration of breast cancer (BC). **(A,B)** miR-551b was relatively low expressed in breast cancer tissues and cells; **(C)** CCK8 experiments showed that cell proliferation was significantly enhanced or inhibited at 48 h and 72 h after interfering or overexpressing miR-551b in MDA-MB-231 cells, respectively; **(D,E)** Transwell experiments showed that the interference or overexpression of miR-551b invasive ability of MDA-MB-231 cells was significantly enhanced or inhibited; **(F,G)** Scratch assay showed that the migration ability of breast cancer cells was significantly enhanced or inhibited after interfering or overexpressing miR-551b in MDA-MB-231.

We verified miR-551b expression in BC tissues and cells, as well as its effects on cancer proliferation, invasion, and migration, to identify the involvement of miR-551b in the expression and progression of BC. Initially, we found miR-551b expression in the paired normal tissues and BC tissues. Figure 6A demonstrates that miR-551b expression was comparatively low in BC tissues and BC cells. This is in line with our earlier findings genetically. Moreover, miR-551b expression is lower in BC cells than it is in normal cells (Figure 6B). Additionally, we transfected miR-551b mimics and antagonists into MDA-MB-231 cells and used the CCK8 assay, Transwell assay, and scratch test to monitor changes in the ability of cancer cells to proliferate, invade, and migrate (Figures 6C–G). As shown in the results, the activity of cancer cells was dramatically raised or lowered at 48 h and 72 h, after interfering with or overexpressing miR-551b in MDA-MB-231 cells, respectively. It indicated that interfering or overexpressing miR-551b could enhance or inhibit the proliferation ability of cancer cells. The cell counts of MDA-MB-231 crossing the matrix-gel were significantly elevated or decreased after interfering or overexpressing miR-551b, indicating that the invasive ability of cancer cells was significantly elevated or decreased after miR-551b interference or overexpression (Figures 6D, E). After miR-551b inhibition or overexpression, cell healing ability for the migration assay was either dramatically increased or diminished, respectively, indicating that miR-551b could regulate the migration ability of cancer cells (Figures 6F, G). These findings reveal that miR-551b affects BC proliferation, invasion, and migration.

## 4 Discussion

More than 60% of the mRNAs in the human genome are regulated by miRNAs, and miRNA abnormalities play a key role in the development and spread of tumors (Iorio and Croce, 2012). On the one hand, miRNAs regulate target gene expression, which has an impact on the development and spread of tumors. On the other hand, cancer formation and occurrence are brought on by miRNA alterations. MiRNAs, however, play a significant role in metastasis, tumor invasion, cell cycle, and BC proliferation (Yang et al., 2017; Abdalla et al., 2020). Clarifying the dysfunction mechanism of miRNAs in breast cancer is therefore required. For the first time, the function of miRNAs in breast cancer and their regulation mechanisms were thoroughly explained in this work.

Although there have been studies on the prognostic roles of microRNAs, only a small number of miRNAs comprehensively focus on breast cancer BC OS and DSS (Hill et al., 2018; Song et al., 2022). The indispensable roles of DSS, enable us to monitor cancer development and deterioration, to timely intervention and treatment (Tahmassebi et al., 2019; Miao et al., 2020). In the present study, we originally explored the effect of DSS on the BC prognosis, and constructed a risk model based on miRNAs DSS, and validated it its roles in BC OS. Notably, miRNAs constructed in each model differ in survival outcomes, which highlighted the limitation of OS on prognosis evaluation. Therefore, it is necessary to comprehensively evaluate BC endpoint outcomes such as DSS and OS.

Through the comprehensive assessment of prognostic endpoint events in BC, we finally confirmed that has-miR-551b was a significant miRNA for BC prognosis. miR-551b is located on chromosome 3q26.2 and participates in a variety of biological processes, such as inflammatory reaction, carcinogenic, chemoresistance (Chaluvally-Raghavan et al., 2014; Wei et al., 2016; Zhang et al., 2018). In thyroid cancers, miR-551b expression levels were correlated with lymph node metastasis, and TNM stage (Dong et al., 2023). And miR-551b can be an independent prognostic factor with lower overall survival and worse prognosis of lung adenocarcinoma (Lin et al., 2016). In the chemotherapy resistance of lung cancer, miR-551b promotes the sensitivity of cells to apoptotic toxicity induced by chemotherapeutic agents by reducing the expression of cell catalase, inhibiting the accumulation of reactive oxygen species and the expression of MUC1 protein. Downregulation of miR-551b can upregulate the expression of cellular catalase, promote the accumulation of reactive oxygen species, and upregulate the expression of MUC1 protein, and the miR-551b/catalase/ROS/MUC1 protein pathway can be used as a target for treatment of acquired chemotherapy resistance (Kharbanda et al., 2014; Xu et al., 2014). The miR-551b was identified as downregulated miRNAs in gastric cancer (GC) the microarray and validated in the research of Chen et al. (2014), Chen et al. (2015). And the expression of miR-551b was low in GC cells, which could regulate epithelial-mesenchymal transition and metastasis via inhibiting ERBB4 expression (Song et al., 2017). Moreover, miR-551b suppresses the expression of TRIM31, thereby inhibiting the proliferation, migration, oxidative stress and apoptosis of MDA-MB-231 cells (Yang et al., 2022).

In this research, we identified miRNAs related to DSS and OS. Through TCGA_DSS, TCGA_OS, AND DSS validation groups, we confirmed the protective factor of miR-551b in BC prognosis. Additionally, we validated our results in METABRIC, TCGA, and GSE19783 datasets. And tissue and cell verification also backen our findings. These findings imply that miR-551b’s aberrant expression plays a significant role in the development and spread of tumors. But the research on miR-551b in BC is limited. Here, we comprehensively investigated DEMs expression in BC and its prognostic impact. By the validation of different endpoint event risk models, we identified miR-551b as an independent protective factor for BC prognosis. Additional research indicates that miR-551b is a viable target for BC treatment because of it is effect on BC proliferation, invasion, and migration.

## Data Availability

The datasets presented in this study can be found in online repositories. The names of the repository/repositories and accession number(s) can be found in the article/Supplementary Material.

## Supplementary material

SUPPLEMENTARY FIGURE S1 Validation of the prognostic effect of DSS-related DEMs in breast cancer (BC). **(A–D)** Validating the prognostic effect of DSS-related DEMs, including hsa-let-7b, hsa-mir-205, hsa-mir-381, and hsa-mir-410, in the TCGA database, respectively. **(E)** A prognostic-related nomogram was constructed by qualified DEMs for multivariate analysis. **(F–H)** Correction curves assessing the theoretical and practical predictive ability of prognostic risk at 1, 3, and 5 years.

SUPPLEMENTARY FIGURE S2 Prognostic evaluation of risk model involved in OS-related DEMs. **(A–F)** Prognostic value of hsa-let-7b, hsa-mir-30a, hsa-mir-103a.1, hsa-mir-449a, hsa-mir-551b, hsa-mir-1247, in BC-OS of TCGA database, respectively.

SUPPLEMENTARY FIGURE S3 Identification of BC-related DEMs in the OS validation group. **(A)** Univariate Cox analysis validating the prognostic effect of OS-related DEMs in OS validation group; **(B)** Lasso regression optimizing the univariate results and including the best variables into the multivariate analysis; **(C)** Multivariate Cox analysis confirming qualified DEMs for risk model. **(D)** KM survival analysis assessed the difference in survival between high- and low-risk patients. **(E)** The risk model classified BC patients into high and low-risk groups according to OS score. **(F)** ROC curves validated the predictive ability of the risk model at 1, 3, and 5 years, respectively.

SUPPLEMENTARY FIGURE S4 Prognostic validation of final qualified miRNAs both in DSS and **(A–C) **OS Prognostic value of hsa-mir-449a in OS of TCGA, METABRIC and GSE40267, respectively. **(D–F)** Prognostic value of hsa-mir-551b in OS of TCGA, METABRIC, and GSE19783 databases, respectively.

SUPPLEMENTARY TABLE S1 All the raw data of three groups including the DSS group, OS group, and DSS validation group, respectively.

SUPPLEMENTARY TABLE S2 The code used in R software was provided in table.

SUPPLEMENTARY TABLE S3 Functional analysis of miR-551b.
