# Correction: A comprehensive analysis of the prognostic characteristics of microRNAs in breast cancer

**DOI:** 10.3389/fgene.2026.1749292

**Published:** 2026-03-11

**Authors:** Lingying Wang, Gui Wang, Jiahong Song, Di Yao, Yong Wang, Tianyou Chen

**Affiliations:** 1 Department of Thoracic Surgery, Wuhan Third Hospital, Tongren Hospital of Wuhan University, Wuhan, China; 2 Department of General Surgery, The Second Affiliated Hospital of Anhui Medical University, Hefei, China

**Keywords:** breast cancer, overall survival, disease specific survival, microRNA-551b, cancer progression

There was a mistake in Figure 6 as published. The authors misused the images of NC, antagomiR-551b and agomiR-551b in Figure 6D. The corrected Figure 6 appears below.

**FIGURE 6:**
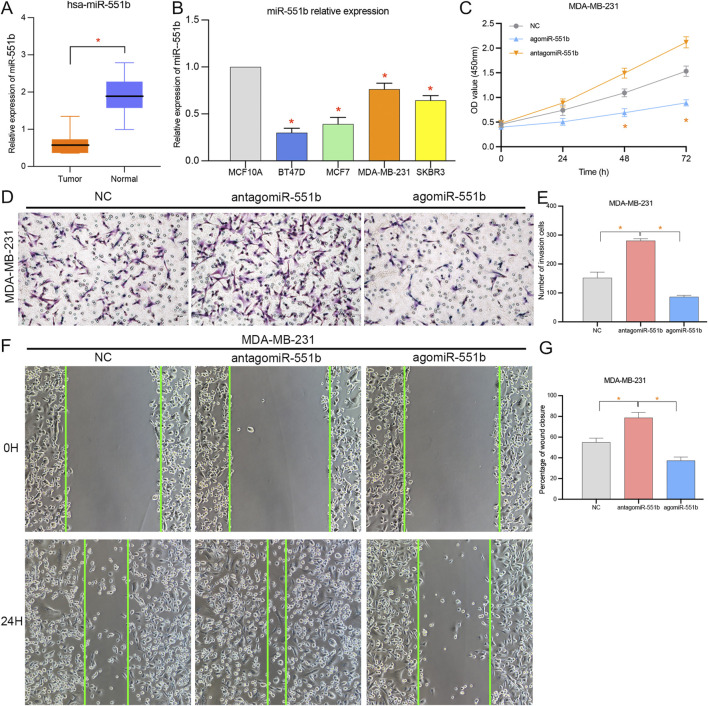
Effects of miR-551b on the proliferation, invasion, and migration of breast cancer (BC). **(A,B)** miR-551b was relatively low expressed in breast cancer tissues and cells; **(C)** CCK8 experiments showed that cell proliferation was significantly enhanced or inhibited at 48 and 72 h after interfering or overexpressing miR-551b in MDA-MB-231 cells, respectively; **(D,E)** Transwell experiments showed that the interference or overexpression of miR-551b invasive ability of MDA-MB-231 cells was significantly enhanced or inhibited; **(F,G)** Scratch assay showed that the migration ability of breast cancer cells was significantly enhanced or inhibited after interfering or overexpressing miR-551b in MDA-MB-231.

The original article has been updated.

