# Supplementary figures and images for: A comprehensive analysis of the prognostic characteristics of microRNAs in breast cancer
Source: Front Genet. 2024 Mar 20;15:1293824. doi: 10.3389/fgene.2024.1293824 (PMC10987719; doi:10.3389/fgene.2024.1293824)

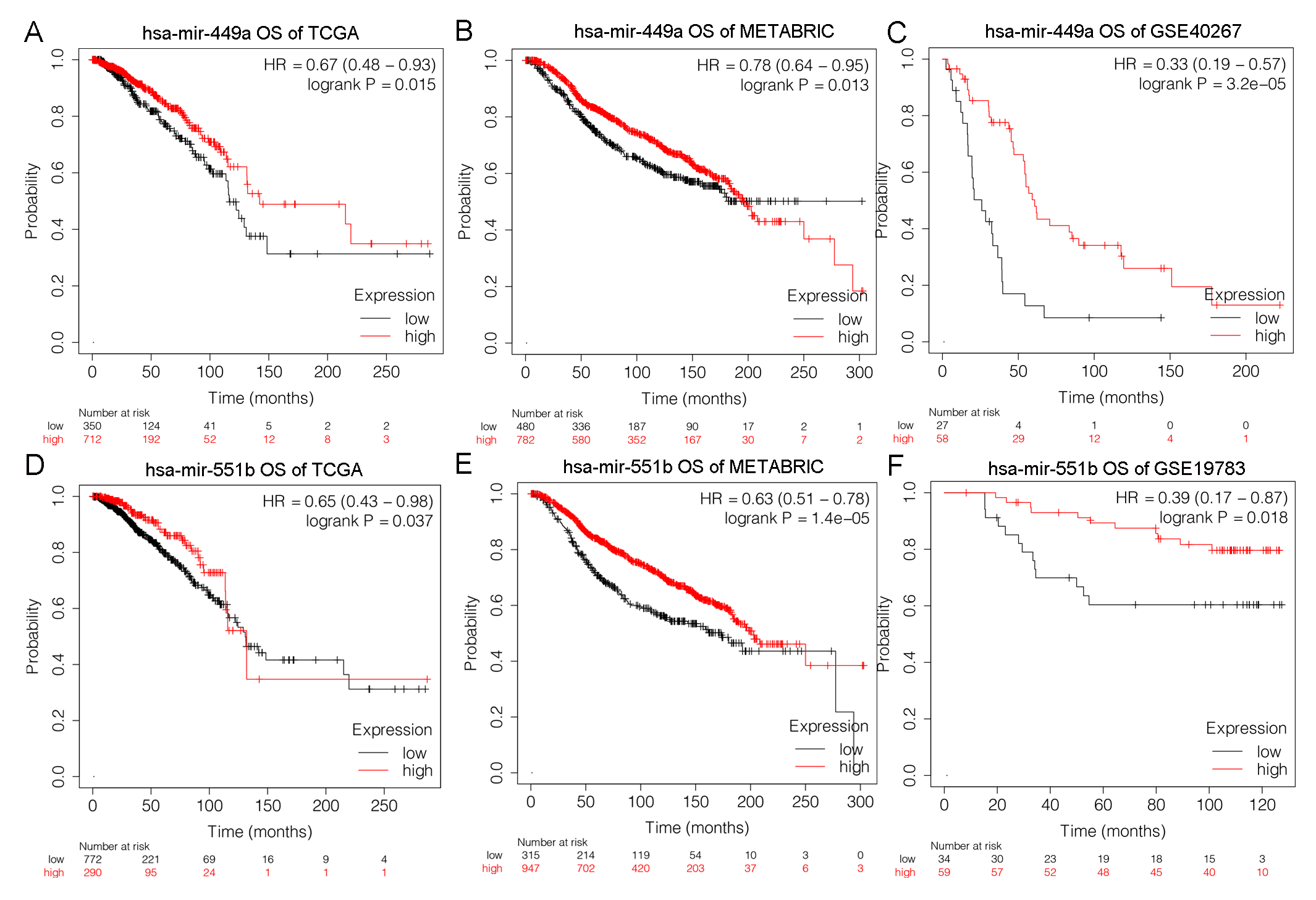

Supplement: Supplementary file 2 [file Figure10.TIF]

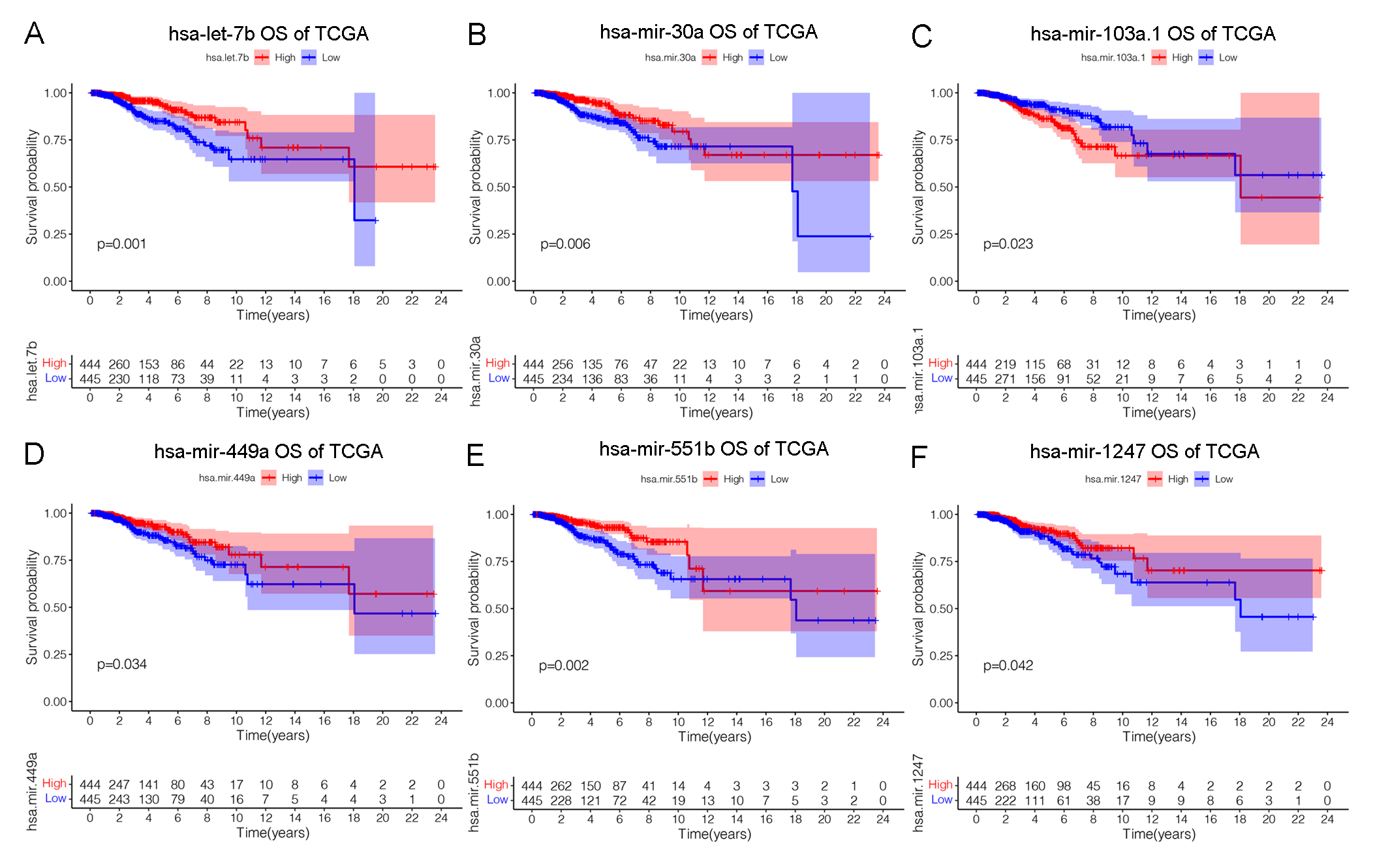

Supplement: Supplementary file 3 [file Figure8.TIF]

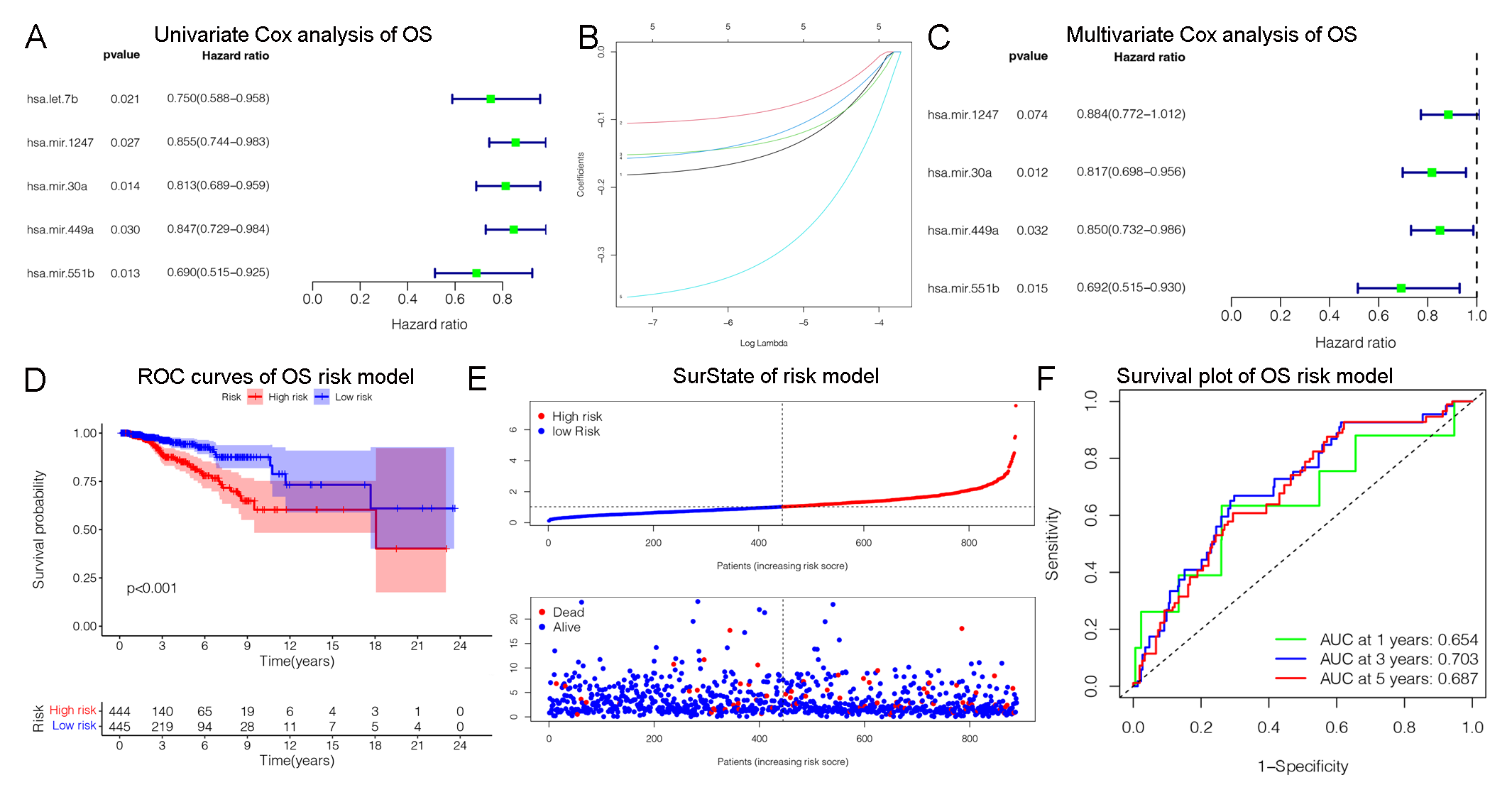

Supplement: Supplementary file 6 [file Figure9.TIF]

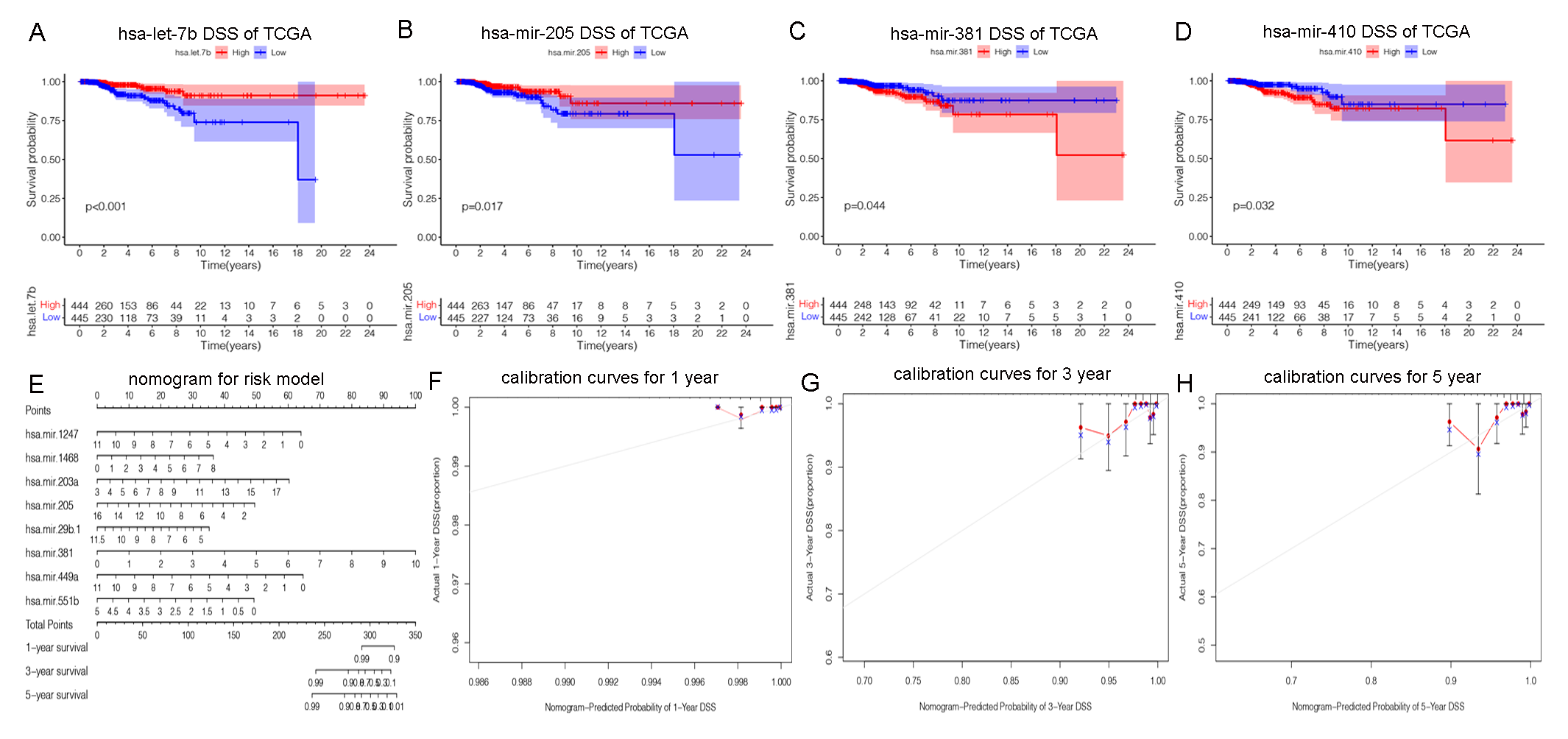

Supplement: Supplementary file 7 [file Figure7.TIF]
