# Supplementary material for: A comprehensive analysis of the prognostic characteristics of microRNAs in breast cancer
Source: Front Genet. 2024 Mar 20;15:1293824. doi: 10.3389/fgene.2024.1293824 (PMC10987719; doi:10.3389/fgene.2024.1293824)
Supplement: Supplementary file 4 [file DataSheet1.PDF]

Firgure 1-4, S1, S3

```
> ###Step1: Uni cox analysis
> rm(list=ls())
> library(survival)
> pFilter=0.05
>
rt=read.table("f1_DSS.txt",header=T,sep="\t
",check.names=F,row.names=1)
> outTab=data.frame()
> sigGenes=c("DSS_futime","DSS_fustat")
> for(i in colnames(rt[,3:ncol(rt)])){
+ cox <- coxph(Surv(DSS_futime,
DSS_fustat) ~ rt[,i], data = rt)
+ coxSummary = summary(cox)
+
coxP=coxSummary$coefficients[,"Pr(>|z|)"]
+ if(coxP<pFilter){
+ sigGenes=c(sigGenes,i)
+ outTab=rbind(outTab,
+ cbind(id=i,
+
HR=coxSummary$conf.int[,"exp(coef)"],
+
HR.95L=coxSummary$conf.int[,"lower .95"],
+
HR.95H=coxSummary$conf.int[,"upper .95"],
+
pvalue=coxSummary$coefficients[,"Pr(>|z|)"
])
+ )
+ }
+ }
write.table(outTab,file="uniCox_DSS.txt",sep
="\t",row.names=F,quote=F)
> uniSigExp=rt[,sigGenes]
>
uniSigExp=cbind(id=row.names(uniSigExp),
uniSigExp)
>
write.table(uniSigExp,file="uniSigExp_DSS.tx
t",sep="\t",row.names=F,quote=F)
> ######Output forest map
> rt <-
read.table("uniCox_DSS.txt",header=T,sep=
"\t",row.names=1,check.names=F)
> gene <- rownames(rt)
> hr <- sprintf("%.3f",rt$"HR")
> hrLow  <- sprintf("%.3f",rt$"HR.95L")
> hrHigh <- sprintf("%.3f",rt$"HR.95H")
> Hazard.ratio <- paste0(hr,"(",hrLow,"-
",hrHigh,")")
> pVal <- ifelse(rt$pvalue<0.001, "<0.001",
sprintf("%.3f", rt$pvalue))
> ###Forest map parameter
> pdf(file="Uniforest_DSS.pdf", width =
7,height = 4)
> n <- nrow(rt)
> nRow <- n+1
> ylim <- c(1,nRow)
> layout(matrix(c(1,2),nc=2),width=c(3,2.5))
> ###plot left parameter
> xlim = c(0,3)
> par(mar=c(4,2.5,2,1))
>
plot(1,xlim=xlim,ylim=ylim,type="n",axes=F,
xlab="",ylab="")
> text.cex=0.8
> text(0,n:1,gene,adj=0,cex=text.cex)
> text(1.5-
0.5*0.2,n:1,pVal,adj=1,cex=text.cex);text(1.5
-
0.5*0.2,n+1,'pvalue',cex=text.cex,font=2,adj
=1)
>
text(3,n:1,Hazard.ratio,adj=1,cex=text.cex);t
ext(3,n+1,'Hazard
ratio',cex=text.cex,font=2,adj=1)
> ###plot right parameter
> par(mar=c(4,1,2,1),mgp=c(2,0.5,0))
> xlim =
c(0,max(as.numeric(hrLow),as.numeric(hrHi
gh)))
>
plot(1,xlim=xlim,ylim=ylim,type="n",axes=F,
ylab="",xaxs="i",xlab="Hazard ratio")
>
arrows(as.numeric(hrLow),n:1,as.numeric(hr
High),n:1,angle=90,code=3,length=0.05,col
```

```
="darkblue",lwd=2.5)
> abline(v=1,col="black",lty=2,lwd=2)
> boxcolor = ifelse(as.numeric(hr) > 1, 'red',
'green')
> points(as.numeric(hr), n:1, pch = 15, col =
boxcolor, cex=1.3)
> axis(1)
> dev.off()
pdf
  2
>
>
> ###Step2: lasso regression analysis
> rm(list = ls())
> library(glmnet)
> library(survival)
> inputfile="uniSigExp_DSS.txt"
> rt<-
read.table(inputfile,header=T,sep="\t",row.
names = 1,check.names =
F,stringsAsFactors = F)
> rtEXP=(rt[,3:ncol(rt)])
> rt=cbind(rt[,1:2],rtEXP)
> rt[,"DSS_futime"]=rt[,"DSS_futime"]/365
> v1<-as.matrix(rt[,c(3:ncol(rt))])
> v2 <-
as.matrix(Surv(rt$DSS_futime,rt$DSS_fustat))
>
> myfit <- glmnet(v1, v2, family = "cox")
> pdf("lambda_DSS.pdf")
> plot(myfit, xvar = "lambda", label = TRUE)
> dev.off()
pdf
  2
> myfit2 <- cv.glmnet(v1, v2, family="cox")
> pdf("min_DSS.pdf")
> plot(myfit2)
>
abline(v=log(c(myfit2$lambda.min,myfit2$l
ambda.1se)),lty="dashed")
> dev.off()
pdf
  2
>
> myfit2$lambda.min
[1] 0.003303489
> coe <- coef(myfit, s = myfit2$lambda.min)
> act_index <- which(coe != 0)
> act_coe <- coe[act_index]
> row.names(coe)[act_index]
 [1] "hsa.let.7b" "hsa.mir.1247"
"hsa.mir.128.1" "hsa.mir.1468"
"hsa.mir.203a" "hsa.mir.205"
 [7] "hsa.mir.29b.1" "hsa.mir.328"
"hsa.mir.340" "hsa.mir.378a"
"hsa.mir.381" "hsa.mir.410"
[13] "hsa.mir.421" "hsa.mir.449a"
"hsa.mir.551b"
> ###Step3: Multivariate Cox analysis
> rm(list=ls())
> library(survival)
>
rt=read.table("uniSigExp_DSS.txt",header=T
,sep="\t",check.names=F,row.names=1)
> rt$DSS_futime=rt$DSS_futime/365
> multiCox=coxph(Surv(DSS_futime,
DSS_fustat) ~ ., data = rt)
> multiCox=step(multiCox,direction =
"both")
Start:  AIC=417.66
Surv(DSS_futime, DSS_fustat) ~ hsa.let.7b +
hsa.mir.1247 + hsa.mir.128.1 +
    hsa.mir.1468 + hsa.mir.203a +
hsa.mir.205 + hsa.mir.29b.1 +
    hsa.mir.328 + hsa.mir.340 +
hsa.mir.378a + hsa.mir.381 +
    hsa.mir.410 + hsa.mir.421 +
hsa.mir.449a + hsa.mir.551b

                Df    AIC
- hsa.mir.340    1 415.66
- hsa.mir.410    1 415.67
- hsa.mir.378a   1 415.70
- hsa.mir.421    1 415.71
- hsa.mir.128.1  1 415.87
- hsa.mir.328    1 416.34
- hsa.let.7b     1 416.76
<none>             417.66
```

- hsa.mir.29b.1 1 418.14
- hsa.mir.381 1 418.61
- hsa.mir.1468 1 418.85
- hsa.mir.551b 1 421.98
- hsa.mir.205 1 423.61
- hsa.mir.449a 1 423.67
- hsa.mir.1247 1 424.35
- hsa.mir.203a 1 424.85

Step: AIC=415.66
Surv(DSS_futime, DSS_fustat) ~ hsa.let.7b + hsa.mir.1247 + hsa.mir.128.1 +
hsa.mir.1468 + hsa.mir.203a + hsa.mir.205 + hsa.mir.29b.1 +
hsa.mir.328 + hsa.mir.378a + hsa.mir.381 + hsa.mir.410 +
hsa.mir.421 + hsa.mir.449a + hsa.mir.551b

Df AIC
- hsa.mir.410 1 413.67
- hsa.mir.378a 1 413.70
- hsa.mir.421 1 413.71
- hsa.mir.128.1 1 413.87
- hsa.mir.328 1 414.34
- hsa.let.7b 1 414.88
<none> 415.66
- hsa.mir.29b.1 1 416.33
- hsa.mir.381 1 416.68
- hsa.mir.1468 1 416.85
+ hsa.mir.340 1 417.66
- hsa.mir.551b 1 420.12
- hsa.mir.205 1 421.64
- hsa.mir.449a 1 421.80
- hsa.mir.1247 1 422.38
- hsa.mir.203a 1 422.91

Step: AIC=413.67
Surv(DSS_futime, DSS_fustat) ~ hsa.let.7b + hsa.mir.1247 + hsa.mir.128.1 +
hsa.mir.1468 + hsa.mir.203a + hsa.mir.205 + hsa.mir.29b.1 +
hsa.mir.328 + hsa.mir.378a + hsa.mir.381 + hsa.mir.421 +
hsa.mir.449a + hsa.mir.551b

Df AIC
- hsa.mir.378a 1 411.71
- hsa.mir.421 1 411.72
- hsa.mir.128.1 1 411.91
- hsa.mir.328 1 412.37
- hsa.let.7b 1 412.88
<none> 413.67
- hsa.mir.29b.1 1 414.80
- hsa.mir.1468 1 414.85
+ hsa.mir.410 1 415.66
+ hsa.mir.340 1 415.67
- hsa.mir.551b 1 418.12
- hsa.mir.449a 1 419.81
- hsa.mir.205 1 419.97
- hsa.mir.1247 1 420.42
- hsa.mir.203a 1 421.05
- hsa.mir.381 1 422.25

Step: AIC=411.71
Surv(DSS_futime, DSS_fustat) ~ hsa.let.7b + hsa.mir.1247 + hsa.mir.128.1 +
hsa.mir.1468 + hsa.mir.203a + hsa.mir.205 + hsa.mir.29b.1 +
hsa.mir.328 + hsa.mir.381 + hsa.mir.421 + hsa.mir.449a +
hsa.mir.551b

Df AIC
- hsa.mir.421 1 409.80
- hsa.mir.128.1 1 409.99
- hsa.mir.328 1 410.44
- hsa.let.7b 1 410.90
<none> 411.71
- hsa.mir.29b.1 1 412.82
- hsa.mir.1468 1 413.06
+ hsa.mir.378a 1 413.67
+ hsa.mir.410 1 413.70
+ hsa.mir.340 1 413.71
- hsa.mir.551b 1 416.12
- hsa.mir.205 1 417.97
- hsa.mir.1247 1 418.44
- hsa.mir.449a 1 418.58

- hsa.mir.203a 1 419.64
- hsa.mir.381 1 421.01

Step: AIC=409.8
Surv(DSS_futime, DSS_fustat) ~ hsa.let.7b + hsa.mir.1247 + hsa.mir.128.1 +
hsa.mir.1468 + hsa.mir.203a + hsa.mir.205 + hsa.mir.29b.1 +
hsa.mir.328 + hsa.mir.381 + hsa.mir.449a + hsa.mir.551b

	Df	AIC
- hsa.mir.128.1	1	408.38
- hsa.mir.328	1	408.49
- hsa.let.7b	1	409.18
<none>		409.80
- hsa.mir.29b.1	1	410.86
- hsa.mir.1468	1	411.33
+ hsa.mir.421	1	411.71
+ hsa.mir.378a	1	411.72
+ hsa.mir.340	1	411.77
+ hsa.mir.410	1	411.78
- hsa.mir.551b	1	414.21
- hsa.mir.205	1	416.19
- hsa.mir.1247	1	416.44
- hsa.mir.449a	1	417.19
- hsa.mir.203a	1	417.65
- hsa.mir.381	1	419.23

Step: AIC=408.38
Surv(DSS_futime, DSS_fustat) ~ hsa.let.7b + hsa.mir.1247 + hsa.mir.1468 +
hsa.mir.203a + hsa.mir.205 + hsa.mir.29b.1 + hsa.mir.328 +
hsa.mir.381 + hsa.mir.449a + hsa.mir.551b

	Df	AIC
- hsa.mir.328	1	406.77
- hsa.let.7b	1	407.91
<none>		408.38
- hsa.mir.29b.1	1	409.56
+ hsa.mir.128.1	1	409.80
+ hsa.mir.421	1	409.99
+ hsa.mir.378a	1	410.10
+ hsa.mir.340	1	410.11
+ hsa.mir.410	1	410.26
- hsa.mir.1468	1	410.71
- hsa.mir.551b	1	412.76
- hsa.mir.1247	1	415.24
- hsa.mir.449a	1	415.54
- hsa.mir.205	1	415.93
- hsa.mir.203a	1	416.24
- hsa.mir.381	1	419.24

Step: AIC=406.77
Surv(DSS_futime, DSS_fustat) ~ hsa.let.7b + hsa.mir.1247 + hsa.mir.1468 +
hsa.mir.203a + hsa.mir.205 + hsa.mir.29b.1 + hsa.mir.381 +
hsa.mir.449a + hsa.mir.551b

	Df	AIC
- hsa.let.7b	1	406.40
<none>		406.77
+ hsa.mir.328	1	408.38
- hsa.mir.29b.1	1	408.42
+ hsa.mir.128.1	1	408.49
+ hsa.mir.378a	1	408.54
+ hsa.mir.421	1	408.55
+ hsa.mir.340	1	408.55
+ hsa.mir.410	1	408.65
- hsa.mir.1468	1	408.73
- hsa.mir.551b	1	412.94
- hsa.mir.1247	1	413.46
- hsa.mir.449a	1	414.08
- hsa.mir.205	1	414.44
- hsa.mir.203a	1	414.77
- hsa.mir.381	1	418.49

Step: AIC=406.4
Surv(DSS_futime, DSS_fustat) ~ hsa.mir.1247 + hsa.mir.1468 +
hsa.mir.203a + hsa.mir.205 + hsa.mir.29b.1 + hsa.mir.381 +
hsa.mir.449a + hsa.mir.551b

	Df	AIC

```
<none>                    406.40
+ hsa.let.7b       1 406.77
+ hsa.mir.340      1 407.64
+ hsa.mir.328      1 407.91
+ hsa.mir.421      1 407.92
+ hsa.mir.128.1    1 408.02
- hsa.mir.29b.1    1 408.11
+ hsa.mir.378a     1 408.14
+ hsa.mir.410      1 408.31
- hsa.mir.1468     1 408.91
- hsa.mir.551b     1 412.66
- hsa.mir.449a     1 413.40
- hsa.mir.203a     1 414.20
- hsa.mir.205      1 415.02
- hsa.mir.1247     1 415.88
- hsa.mir.381      1 419.28
> multiCoxSum=summary(multiCox)
> outTab=data.frame()
> outTab=cbind(
+
coef=multiCoxSum$coefficients[,"coef"],
+ HR=multiCoxSum$conf.int[,"exp(coef)"],
+HR.95L=multiCoxSum$conf.int[,"lower .95
"],+HR.95H=multiCoxSum$conf.int[,"upper
.95"],+pvalue=multiCoxSum$coefficients[,"
Pr(>|z|)"])
>
outTab=cbind(id=row.names(outTab),outT
ab)
> outTab=gsub("`","",outTab)
>
write.table(outTab,file="multiCox_DSS.txt",s
ep="\t",row.names=F,quote=F)
>
riskScore=predict(multiCox,type="risk",new
data=rt)
>
coxGene=rownames(multiCoxSum$coeffici
ents)
> coxGene=gsub("`","",coxGene)
>
outCol=c("DSS_futime","DSS_fustat",coxGe
ne)
>
risk=as.vector(ifelse(riskScore>median(riskS
core),"high","low"))
>
write.table(cbind(id=rownames(cbind(rt[,ou
tCol],riskScore,risk)),cbind(rt[,outCol],riskSco
re,risk)),
+                file="risk_DSS.txt",
+                sep="\t",
+                quote=F,
+                row.names=F)
> ###Output forest map
>                 rt                <-
read.table("multiCox_DSS.txt",header=T,sep
="\t",row.names=1,check.names=F)
> gene <- rownames(rt)
> hr <- sprintf("%.3f",rt$"HR")
> hrLow  <- sprintf("%.3f",rt$"HR.95L")
> hrHigh <- sprintf("%.3f",rt$"HR.95H")
>  Hazard.ratio  <-  paste0(hr,"(",hrLow,"-
",hrHigh,")")
> pVal <- ifelse(rt$pvalue<0.001, "<0.001",
sprintf("%.3f", rt$pvalue))
> ###Forest map parameter
>  pdf(file="multiforest_DSS.pdf",  width  =
7,height = 4)
> n <- nrow(rt)
> nRow <- n+1
> ylim <- c(1,nRow)
> layout(matrix(c(1,2),nc=2),width=c(3,2.5))
> xlim = c(0,3)
> par(mar=c(4,2.5,2,1))
>
plot(1,xlim=xlim,ylim=ylim,type="n",axes=F,
xlab="",ylab="")
> text.cex=0.8
> text(0,n:1,gene,adj=0,cex=text.cex)
>                                  text(1.5-
0.5*0.2,n:1,pVal,adj=1,cex=text.cex);text(1.5
-
0.5*0.2,n+1,'pvalue',cex=text.cex,font=2,adj
=1)
>
text(3,n:1,Hazard.ratio,adj=1,cex=text.cex);t
ext(3,n+1,'Hazard
```

```
ratio',cex=text.cex,font=2,adj=1)
> par(mar=c(4,1,2,1),mgp=c(2,0.5,0))
> xlim = c(0,max(as.numeric(hrLow),as.numeric(hrHigh)))
> plot(1,xlim=xlim,ylim=ylim,type="n",axes=F,ylab="",xaxs="i",xlab="Hazard ratio")
> arrows(as.numeric(hrLow),n:1,as.numeric(hrHigh),n:1,angle=90,code=3,length=0.05,col="darkblue",lwd=2.5)
> abline(v=1,col="black",lty=2,lwd=2)
> boxcolor = ifelse(as.numeric(hr) > 1, 'red', 'green')
> points(as.numeric(hr), n:1, pch = 15, col = boxcolor, cex=1.3)
> axis(1)
> dev.off()
pdf
  2
>
>
> ###Step4: ROC curve and survival curve are drawn
> rm(list = ls())
> library(survival)
> library(survminer)
> library(timeROC)
> inputFile="risk_DSS.txt"
> survFile="survival_DSS.pdf"
> rocFile="ROC_DSS.pdf"
> rt=read.table(inputFile,header=T,sep="\t")
> #comparing survival difference between groups and P-value
> diff=survdiff(Surv(DSS_futime, DSS_fustat) ~risk,data = rt)
> pValue=1-pchisq(diff$chisq,df=1)
> fit <- survfit(Surv(DSS_futime, DSS_fustat) ~ risk, data = rt)
> if(pValue<0.001){
+     pValue="p<0.001"
+ }else{
+ pValue=paste0("p=",sprintf("%.03f",pValue))
+ }
> #Plot survival curve
> surPlot=ggsurvplot(fit,
+                data=rt,
+                conf.int=T,
+                pval=pValue,
+                pval.size=5,
+                risk.table=TRUE,
+ legend.labs=c("High risk", "Low risk"),
+                legend.title="Risk",
+                xlab="Time(years)",
+                break.time.by = 3,
+                risk.table.title="",
+                palette=c("red", "blue"),
+ risk.table.height=.25)
> pdf(file=survFile,onefile = FALSE,width = 6.5,height =5.5)
> print(surPlot)
> dev.off()
pdf
  2
> ###Plot ROC curve
> ROC_rt=timeROC(T=rt$DSS_futime,delta=rt$DSS_fustat,
+ marker=rt$riskScore,cause=1,
+            weighting='aalen',
+ times=c(1,3,5),ROC=TRUE)
> pdf(file=rocFile,width=5,height=5)
> plot(ROC_rt,time=1,col='green',title=FALSE,lwd=2)
> plot(ROC_rt,time=3,col='blue',add=TRUE,title=FALSE,lwd=2)
> plot(ROC_rt,time=5,col='red',add=TRUE,title=FALSE,lwd=2)
```

```
> legend('bottomright',
+                   c(paste0('AUC at 1 years: ',round(ROC_rt$AUC[1],3)),
+                     paste0('AUC at 3 years: ',round(ROC_rt$AUC[2],3)),
+                     paste0('AUC at 5 years: ',round(ROC_rt$AUC[3],3))),
+ col=c("green",'blue','red'),lwd=2,bty = 'n')
> dev.off()
pdf
  2
>
> ###plot heatmap
> rm(list = ls())
> library(pheatmap)
> rt=read.table("risk_DSS.txt",sep="\t",header=T,row.names=1,check.names=F)
> rt=rt[order(rt$riskScore),]
> riskClass=rt[,"risk"]
> lowLength=length(riskClass[riskClass=="low"])
> highLength=length(riskClass[riskClass=="high"])
> line=rt[,"riskScore"]
> line[line>10]=10
> pdf(file="riskScore_DSS.pdf",width = 10,height = 4)
> plot(line,
+      type="p",
+      pch=20,
+      xlab="Patients (increasing risk socre)",
+      ylab="Risk score",
+      col=c(rep("blue",lowLength),
+            rep("red",highLength)))
> abline(h=median(rt$riskScore),v=lowLength,lty=2)
> legend("topleft", c("High risk", "low Risk"),bty="n",pch=19,col=c("red","blue"),cex=1.3)
> dev.off()
pdf
  2
> color=as.vector(rt$DSS_fustat)
> color[color==1]="red"
> color[color==0]="blue"
> pdf(file="survStat_DSS.pdf",width = 10,height = 4)
> plot(rt$DSS_futime,
+      pch=19,
+      xlab="Patients (increasing risk socre)",
+      ylab="Survival time (years)",
+      col=color)
> legend("topleft", c("Dead", "Alive"),bty="n",pch=19,col=c("red","blue"),cex=1.3)
> abline(v=lowLength,lty=2)
> dev.off()
pdf
  2
>

version.string R version 4.3.1 (2023-06-16)
nickname       Beagle Scouts
> packageVersion('survival')
[1] '3.5.7'
> packageVersion('survminer')
[1] '0.4.9'
> packageVersion('glmnet')
[1] '4.1.8'
> packageVersion('timeROC')
[1] '0.4'
> packageVersion('pheatmap')
[1] '1.0.12'
```

Firgure S1 E-H

```
> rm(list = ls())
> riskFile="risk_DSS.txt"
> outFile="Nomogram_DSS.pdf"
>risk=read.table(riskFile,header=T,sep="\t", check.names=F,row.names=1)
> rt=risk[,1:(ncol(risk)-2)]
> dd <- datadist(rt)
> options(datadist="dd")
> f <- cph(Surv(DSS_futime, DSS_fustat) ~ hsa.mir.1247 + hsa.mir.1468 + hsa.mir.203a + hsa.mir.205 +
+ hsa.mir.29b.1 + hsa.mir.381 + hsa.mir.449a + hsa.mir.551b, x=T, y=T, surv=T, data=rt, time.inc=1)
> surv <- Survival(f)
> nomogram
> nom <- nomogram(f, fun=list(function(x) surv(1, x), function(x) surv(3, x), function(x) surv(5, x)),
+ lp=F, funlabel=c("1-year survival", "3-year survival", "5-year survival"),
+ maxscale=100,
+ fun.at=c(0.99, 0.9, 0.8, 0.7, 0.5, 0.3,0.1,0.01))
> #nomogram 可视化
> pdf(file=outFile,height=6,width=9)
> plot(nom)
> dev.off()
pdf
  2
> #calibration curve for 1 year
> time=1
> f <- cph(Surv(DSS_futime, DSS_fustat) ~ hsa.mir.1247 + hsa.mir.1468 + hsa.mir.203a + hsa.mir.205 +
+ hsa.mir.29b.1 + hsa.mir.381 + hsa.mir.449a + hsa.mir.551b, x=T, y=T, surv=T, data=rt, time.inc=time)
> cal <- calibrate(f, cmethod="KM", method="boot", u=time, m=80, B=1000)
Using Cox survival estimates at  1 Days
There were 50 or more warnings (use warnings() to see the first 50)
>pdf(file="calibration 1 year.pdf",height=6,width=8)
> plot(cal,xlab="Nomogram-Predicted Probability of 1-Year DSS",ylab="Actual 1-Year DSS(proportion)",col="red",sub=F)
> dev.off()
pdf
> #calibration curve for 3 year
> time=3
> f <- cph(Surv(DSS_futime, DSS_fustat) ~ hsa.mir.1247 + hsa.mir.1468 + hsa.mir.203a + hsa.mir.205 +
+ hsa.mir.29b.1 + hsa.mir.381 + hsa.mir.449a + hsa.mir.551b, x=T, y=T, surv=T, data=rt, time.inc=time)
> cal <- calibrate(f, cmethod="KM", method="boot", u=time, m=80, B=1000)
Using Cox survival estimates at  3 Days
> pdf(file="calibration 3 year.pdf",height=6,width=8)
> plot(cal,xlab="Nomogram-Predicted Probability of 3-Year DSS",ylab="Actual 3-Year DSS(proportion)",col="red",sub=F)
> dev.off()
pdf
> #calibration curve for 5 year
> time=5
> f <- cph(Surv(DSS_futime, DSS_fustat) ~ hsa.mir.1247 + hsa.mir.1468 + hsa.mir.203a + hsa.mir.205 +
+ hsa.mir.29b.1 + hsa.mir.381 + hsa.mir.449a + hsa.mir.551b, x=T, y=T, surv=T, data=rt, time.inc=time)
> cal <- calibrate(f, cmethod="KM", method="boot", u=time, m=80, B=1000)
Using Cox survival estimates at  5 Days
> pdf(file="calibration 5 year.pdf",height=6,width=8)
> plot(cal,xlab="Nomogram-Predicted Probability of 5-Year DSS",ylab="Actual 5-Year DSS(proportion)",col="red",sub=F)
> dev.off()
pdf
```
